# The Antibacterial Activity of *Coriolus versicolor* Methanol Extract and Its Effect on Ultrastructural Changes of *Staphylococcus aureus* and *Salmonella* Enteritidis

**DOI:** 10.3389/fmicb.2016.01226

**Published:** 2016-08-04

**Authors:** Danka Matijašević, Milena Pantić, Božidar Rašković, Vladimir Pavlović, Dunja Duvnjak, Aleksandra Sknepnek, Miomir Nikšić

**Affiliations:** ^1^Institute for Food Technology and Biochemistry, Faculty of Agriculture, University of BelgradeBelgrade, Serbia; ^2^Institute of Animal Sciences, Faculty of Agriculture, University of BelgradeBelgrade, Serbia; ^3^Institute of Agricultural Engineering, Faculty of Agriculture, University of BelgradeBelgrade, Serbia

**Keywords:** antibacterial activity, natural preservatives, foodborne pathogens, mushroom extract, *Coriolus versicolor*, electron microscopy

## Abstract

The antibacterial activity of methanol extract obtained from fruiting body of industrially grown basidiomycete *Coriolus versicolor* was examined. The Minimum Inhibitory Concentration (MIC) values against various bacteria ranged from 0.625 to 20 mg mL^-1^. *C. versicolor* expressed bactericidal activity against both Gram-positive and Gram-negative bacteria. The growth curves of *Staphylococcus aureus* and *Salmonella enterica* serovar Enteritidis, measured at 630 nm, and confirmed with macrodilution method showed that the obtained extract could inhibit the growth of tested bacteria. Scanning electron microscopy (SEM), transmission electron microscopy (TEM), and the loss of 260-nm-absorbing material were used to examine the ultrastructural changes in bacteria induced by the extract. When *S. aureus* was exposed to the MIC of *C. versicolor*, elongated and malformed cells were observed by SEM, while *S.* Enteritidis treated cells appeared shorter and aggregated with ruptured cell walls. TEM revealed the formation of non-membrane-enclosed bodies and depleted inner content of *S. aureus*. Larger and irregular periplasmic space and deformed and scattered components of the cell envelope were observed in treated *S.* Enteritidis. The loss of 260-nm-absorbing material indicated that the disruptive action of the extract on cytoplasmic membrane was more pronounced in *S. aureus* than in *S.* Enteritidis treated cells. The UV and FTIR spectrophotometric analyses revealed diverse composition of *C. versicolor* extract and high content of total phenolics. Altogether, mushroom extracts could be used to develop nutraceuticals or drugs effective against pathogenic microorganisms.

## Introduction

Since ancient times, mushrooms have been recognized as valuable source of nutritive and pharmacologically active compounds. In last decades, extensive studies have been conducted with the aim of examining their antitumorigenic, immunomodulating, antioxidant, cardiovascular, hypolipidemic, detoxifying, hepatoprotective, antidiabetic, and antimicrobial properties (Gao et al., 2003; Rowan et al., 2003; Zhang et al., 2007; Wasser, 2011; Savić et al., 2012). A large number of diverse bioactive compounds have been isolated and identified in mushrooms. Based on the chosen extraction solvent, the type and concentration of bioactive compounds varies in the final extract, which consequently, reflects on the spectrum of their pharmacological activities, including antimicrobial activity. Aqueous extraction is often used for the screening of the antimicrobial potential but, since the aromatic and saturated organic compounds are mainly recognized as active against microorganisms, they are often obtained through methanol or ethanol extraction (Cowan, 1999). A number of studies showed that mushrooms extracts express a higher antimicrobial activity against Gram-positive bacteria. According to literature data, *Staphylococcus aureus, Bacillus cereus*, and *Bacillus subtilis* are the most susceptible bacteria to the inhibitory action of mushrooms. Methanolic extracts of *Agaricus bisporus, Boletus edulis, Cantharellus cibarius* (Barros et al., 2008; Ozen et al., 2011), *Clitocybe alexandri* (Solak et al., 2006), *Lepista nuda* (Dulger et al., 2002), *Coriolus versicolor* (Karaman et al., 2010), *Handkea utriformis, Handkea excipuliformis, Vascellum pratense* (Petrović et al., 2016), *Pleurotus* *ostreatus* (Alves et al., 2012), and different *Lactarius* sp. (Ozen et al., 2011) as well as ethanolic extracts of *Armillaria mellea, Clitocybe geotropa* (Kalyoncu et al., 2008) and *Laetiporus sulphureus* (Turkoglu et al., 2007) demonstrated good inhibitory effects against some or all of the aforementioned bacteria. On the other hand, the activity of different mushrooms and their extracts against Gram-negative bacteria is often scarce and even contradictory results were obtained. Barros et al. (2008) found no activity of *A. bisporus* methanolic extract against Gram-negative bacteria while Tambekar et al. (2006) and Ozen et al. (2011) reported positive activity against *Escherichia coli, Pseudomonas aeruginosa, Proteus vulgaris*, and *Salmonella* Typhimurium. *E. coli* was inhibited by methanolic extracts of *C. alexandri* (Solak et al., 2006), *Lactarius* sp. (Ozen et al., 2011), and *Pleurotus sajor-caju* (Tambekar et al., 2006), while *P. aeruginosa* was sensitive to methanolic extract of *C. alexandri* (Solak et al., 2006), *B. edulis*, and *C. cibarius* (Ozen et al., 2011). Generally, the research was almost entirely focused on the screening of antibacterial properties of mushroom extracts without identifying the compounds responsible for their action. However, some of the identified compounds were sesquiterpenes and other terpenes, steroids, benzoic acid derivatives, anthraquinones and quinolines, as well as peptides and proteins (Alves et al., 2012; Schillaci et al., 2013).

The mechanisms of action of the organic compounds that might be associated with the antimicrobial activity of mushroom extracts have been the subject of many researches. For phenols and phenolic acids, it was evidenced that the site(s) and number of hydroxyl groups determines their relative toxicity to microorganisms (Cowan, 1999; Alves et al., 2013) and the proposed mechanism of their action includes enzyme inhibition by oxidized compounds. Quinones are known to complex irreversibly with nucleophilic amino acids in proteins, leading to inactivation and loss of function of the proteins, which makes them great antimicrobial agents. They possibly interact with cell wall polypeptides, membrane-bound enzymes and surface-exposed adhesins of pathogenic bacteria (Cowan, 1999). Flavonoids as secondary metabolites are synthesized by plants and mushrooms as a response to microbial infections (Ferreyra et al., 2012; Rahi and Malik, 2016). Their activity is linked to their ability to complex with proteins and bacterial cell walls while more lipophilic flavonoids may also disrupt microbial membranes (Tsuchiya et al., 1996). In addition, they may inhibit cell wall, cell membrane and nucleic acid synthesis and energy metabolism (Cushnie and Lamb, 2011). It was suggested that the mode of action of terpenes and their related alcohols involves disruption of microbial membranes by their lipophilic components (Cowan, 1999; Carson et al., 2002). Antimicrobial peptides (AMPs) may act on bacteria in several ways such as the formation of ion channels in the microbial membranes, selective disruption of the cell membranes, activation of molecules in the autolysis cascade within bacterial cells, and inhibition of protein and DNA synthesis (Reddy et al., 2004).

The mode of action of various antimicrobial agents is generally related with interferences in the synthesis of the cell wall, modification of the permeability of plasmatic membrane, interferences in chromosome replication, or in protein synthesis (Alves et al., 2012). Transport, osmoregulation and respiration processes, biosynthesis and cross-linking of peptidoglycan and synthesis of lipids are essential functions regulated by the (inner) bacterial cell membrane. For performing all these functions, membrane integrity is a prerequisite, and its disruption can directly or indirectly cause metabolic dysfunction and cell death (Hartmann et al., 2010). The major function of the cell wall is to provide shape and cell integrity and to act as an osmotic barrier. Hence, observing alterations in bacterial cell structure by electron microscopy (EM) could help to elucidate the mode of action of antimicrobial agents, including mushroom extracts.

The mushroom used in this study has a widespread application as medicinal mushroom, which is consumed in different forms such as food and tea. In recent years, consumers have become concerned about the possible negative health impact of synthetic preservatives used in food. In addition, considerable attention has been given to use of natural antimicrobial compounds due to a drastic increase of antibiotic resistance in foodborne pathogens. This, in turn, has led to a search for antimicrobials derived from natural sources, such as plants, animals, bacteria, algae, and mushrooms, with the aim to reduce the harmful microorganisms and to improve quality and shelf-life of food (Gyawali and Ibrahim, 2014). The incorporation of mushroom extracts as antimicrobial preservatives in food systems has been the subject of research. *Tirmania pinoyi* methanolic extract successfully inhibited the growth of *S. aureus* in chicken soup, kept at room temperature and in refrigerator, in a dose dependent manner (Stojković et al., 2013). Ethanolic extracts of *A. bisporus* and *Agaricus brasiliensis* were efficient in the control of *Listeria monocytogenes* in yogurt (Stojković et al., 2014) while the methanol extract of *Agaricus bohusii* inhibited the development of *Penicillium verrucosum var. cyclopium* in cream cheese (Reis et al., 2012). Complete inhibition of *Aspergillus flavus* growth in tomato paste after 15 days of treatment was achieved with 15 mg mL^-1^ of the methanol extract of *L. sulphureus* (Petrović et al., 2013). Except for proven preserving properties *in situ*, mushroom extracts are also good candidates due to their exquisite taste. Furthermore, there is less chance that microorganisms will become resistant to their effect since they are a complex mixture of different compounds.

*Coriolus versicolor* [*Trametes versicolor* (L.:Fr.) Lloyd, 1920] is a medicinal mushroom with a broad spectrum of physiological activities that has been recognized and used for thousands of years in traditional medicine. Numerous *in vivo* studies revealed that *C. versicolor* extracts have the ability to restore the weakened immune system of cancer patients (Chu et al., 2002). Furthermore, its stimulatory effects on the production of interferon and interleukins were observed in human cells (Sakagami et al., 1992; Chu et al., 2002). Moreover, strong antiviral, significant antioxidant, hepatoprotective and analgesic activities of *Coriolus* spp. fruiting body extracts were reported (Chu et al., 2002; Cui and Chisti, 2003; Sheikh et al., 2014). Antimicrobial activities of *C. versicolor* extract against prevalent pathogens, such as *E. coli, P. aeruginosa, S. aureus, Candida albicans, Klebsiella pneumoniae, L. monocytogenes*, and *Streptococcus pneumoniae*, were noted in some *in vivo* animal studies (Chu et al., 2002). Comparing the solvents, the methanol extracts were in most cases among the strongest antimicrobial agents, whereas water fractions possessed the weakest inhibitory activity (Quereshi et al., 2010; Smolskaitė et al., 2015). The chemical composition of *C. versicolor* extracts is complex and different classes of compounds were suggested as being responsible for their biological activities. Among them, polysaccharide krestin (PSK) and various polysaccharide–peptide complexes as well as terpenoids and polyphenols were mainly considered as responsible for these effects (Chu et al., 2002; Harhaji et al., 2008; Wasser, 2010).

The aim of this study was to determine the chemical composition of methanol extract of *C. versicolor*, using UV and FTIR spectrophotometric analysis, and to examine its antimicrobial potential. The basidiomycete, subject of this study, was grown under controlled conditions which make it suitable for production of nutraceuticals and pharmaceuticals with constant and repeatable quality compared with those found in nature. Furthermore, scanning electron microscopy (SEM), transmission electron microscopy (TEM) and the leakage of the 260-nm-absorbing material were employed to reveal morphological changes of bacterial cells that occurred upon treatment with methanolic mushroom extract. To the best of our knowledge, this is the first study that revealed ultrastructural changes and tried to establish the possible targets of mushroom extracts on bacterial cells.

## Materials and Methods

### Mushroom and Growing Conditions

Pure culture of basidiomycete *Coriolus versicolor* was obtained from the collection of the Department of Industrial Microbiology, Faculty of Agriculture, University of Belgrade. Inoculum (spawn) prepared on wheat grains (Jevtić et al., 2014) was used as seed for fruiting body production. The substrate consisted of oak sawdust, wheat straw, and wheat bran (in 5:3:2 ratio). Bags (Mycelia, Sac O2, Combiness, Belgium) were loaded with moistened (70%) substrate and sterilized at 121°C for 2 h. Upon cooling, 10% inoculums were added to each bag. After the mycelia had completely colonized the substrate in the dark for 20 days, fructification was performed at 20 ± 2°C with 80–95% relative humidity (Growth Chamber GC-1000TLH, Jeio Tech, Korea). The mushrooms were air-dried at 40°C and ground to fine powder (Orth et al., 1993; Mondal et al., 2010; Jevtić et al., 2014).

### Preparation of the Methanol Extract

Mushroom extract was prepared according to a slightly optimized version of the procedure described by Duvnjak et al. (2016). Briefly, 30 g of dried mushroom sample was obtained from 100 g of fresh fruiting body of the *C. versicolor* which had 70% of moisture. A 10 g portion of obtained dried sample was extracted with 150 mL of methanol by stirring at 120 rpm for 24 h at room temperature and filtered through a Whatman No.4 paper. The residue was re-extracted under the same conditions until the extraction solvent remained colorless. Methanol was removed from the combined extracts using a rotary evaporator type R-II (BUCHI, Switzerland) at 40°C to dryness. The extract was stored at 4°C until further use.

### Determination of Extract Composition

The total carbohydrates were measured by the method outlined by DuBois et al. (1956) using D-glucose as the standard reference.

The contents of total and α-glucans were measured using the Yeast and Mushroom β-glucan Assay Kit (Megazyme Int., Wicklow, Ireland), in accordance with the manufacturer’s instructions.

Determination of total protein content was conducted according to the Bradford (1976) method and bovine serum albumin was used to produce the standard calibration curve.

The content of lipids was determined based on the sulfo-phospho-vanillin reaction with cholesterol as the reference (Turlo et al., 2010).

The total soluble phenolics in the extracts were determined using Folin–Ciocalteu reagent with gallic acid as the standard (Kozarski et al., 2012).

The contents of flavonoids were determined by the colorimetric assay described by Jia et al. (1999). (+)-Catechin was used to construct the standard curve. All analyses were performed with the UV-1800 Spectrophotometer (SHIMADZU, Kyoto, Japan).

### FTIR Analysis

The structural characteristics of the methanol extract of *C. versicolor* were identified using ATR-FTIR spectrometer IRAffinity-1 (Shimadzu, Japan). Sample was applied directly on instrument, with no sample preparation prior to spectral measurements (Kazarian and Chan, 2006). For data processing, the software IRsolution was used (Shimadzu, Japan). A background spectrum was taken before the sample spectrum. All measurements were performed in the spectral range 4000–600 cm^-1^ with a resolution of 4 cm^-1^ (Dufour, 2009; Duvnjak et al., 2016).

### Bacterial Strains and Culture Preparation

Five Gram-negative (*Escherichia coli* O157:H7 ATCC 35150, *Salmonella* ser. Enteritidis ATCC 13076, *Shigella sonnei* ATCC 29930, *Yersinia enterocolitica* ATCC 27729, *Proteus hauseri* ATCC 13315) and four Gram-positive bacterial species (*Staphylococcus aureus* ATCC 25923, *Staphylococcus epidermidis* ATCC 12228, *Listeria monocytogenes* ATCC 19111, *Bacillus cereus* ATCC 11778) were used for the antibacterial assay. For the *L. monocytogenes* and *E. coli* O157:H7, Tryptone Soy Broth (TSB)/Agar (TSA; HiMedia Laboratories, India) was used, while for all the other listed strains Müeller Hinton Broth (MHB)/Agar (MHA; HiMedia Laboratories, India) was the medium of choice.

For the antimicrobial testing, 24-h-old bacterial colony was picked and suspended in the appropriate medium (5 mL) under the recommended conditions (aerobically for 20 h at 37°C). The usually used inoculum dose of approximately 10^5^–10^6^ CFU mL^-1^ was achieved by performing serial dilution of each culture and confirmed by the standard plating method.

### Broth Microdilution Assay

Minimum Inhibitory Concentration (MIC) and Minimum Bactericidal Concentration (MBC) assays of mushroom extract were performed by the standard broth microdilution test (Klaus et al., 2015), and modified as described previously (Duvnjak et al., 2016). The concentrations of the mushroom extract ranged from 0.3125 to 40 mg mL^-1^.

### Determination of the Growth Kinetics by Optical Density (OD)

Concentrations ranging from the MIC to the MBC, previously determined by the broth microdilution assay, were used to examine the kinetics of bacterial growth. Samples were prepared in the same manner as for broth microdilution assay, using 96-well plates. MHB served as the negative control, while the appropriate broth with the tested microorganisms served as positive controls. The microplate reader (ELx808, BioTek Instruments, Inc., USA) controlled by Gen5^TM^ Software was used to measure the optical density at a wavelength of 630 nm every hour for 24 h. During the assay, temperature was maintained at 37°C and plate was shaken for 10 s before every reading (Dalgaard and Koutsoumanis, 2001; Li et al., 2010).

### Broth Macrodilution Assay

Kinetic of inactivation was determined by macrodilution method, according to Klaus et al. (2015). The mushroom extract was diluted in 1 mL of bacterial culture in MHB to reach the concentrations ranging from the MIC to the MBC, previously obtained by the broth microdilution assay. Bacterial growth was followed by taking samples at 0, 3, 6, 9, and 24 h and plating on MHA after serial sample dilutions. The bacterial number was counted upon incubation under aerobic conditions, at 37°C for 24 h, and expressed as log_10_ CFU mL^-1^. Positive controls were performed in the same way, except without adding the mushroom extract.

### Loss of 260-nm-Absorbing Material

Loss of 260-nm-absorbing material was determined by the method described by Carson et al. (2002), with some modifications. As the zero point (0 h), bacteria in the mid-exponential growth phase, after 14 h at 37°C in MHB were taken, diluted in a ratio 1:100, and filtered through a 0.22-μm pore size filter (Sartorius, Germany). Mushroom extract was added at a final concentration equivalent to its MIC value and incubated at 37°C. Untreated samples, cells without mushroom extract, were used as control and their incubation was also continued under the same conditions. Additional samples of control and treated cell suspension were taken after 4 and 8 h, diluted and filtered as described above. Filtrates of the appropriate dilution of mushroom extract were prepared and served as blanks. The extracellular 260-nm-absorbing material released from the cells was measured using a Shimadzu UV-1800 UV-VIS Spectrophotometer. The obtained results of the measurements at 260 nm at each time point were expressed as a proportion of the initial OD_260_ value.

### Scanning Electron Microscopy (SEM) Procedure

Examination of the morphological changes of the bacterial cells was performed using SEM (Hartmann et al., 2010; Tyagi et al., 2013). Due to differences in the shape and cell wall structure, *S.* Enteritidis and *S. aureus* were chosen as model microorganisms for testing. Bacteria in the mid-exponential growth phase (14 h at 37°C in MHB) were treated with the mushroom extracts at the MIC level or left untreated as the control. These suspensions were incubated for an additional 8 h at 37°C and then harvested by centrifugation and prefixed with 2.5% glutaraldehyde overnight at 4°C. The obtained cell pellets were washed three times with 0.1 M sodium phosphate buffer (pH 7.2) and subsequently dehydrated with consequently 25, 50, 75, 90, and 100% ethanol. The dehydrated samples were air dried immediately, followed by smearing on SEM stubs and gold-covering by a Baltec scd 005 sputter coater accessory. The micrographs were obtained with a JEOL JSM-6390LV scanning electron microscope (JEOL USA, Inc.).

### Transmission Electron Microscopy (TEM) Procedure

Ultrastructural damage of the bacterial cells was evaluated using TEM (Hartmann et al., 2010; Tyagi et al., 2013). Bacterial suspensions were prepared in the same manner as described above for the SEM samples. Fixed and washed cell pellets were post-fixed in 1% OsO_4_ for 1 h and rinsed with 0.1 M sodium phosphate buffer (pH 7.2). The samples were then dehydrated with a graded ethanol series (25, 50, 75, 90, and 2 × 100%, 15 min each), washed in propylene oxide (PO) and infiltrated with the mixture of PO/Epon resin (1:1) overnight at room temperature. Fresh Epon was changed three times before the samples were embedded in fresh Epon resin and polymerized at 60°C for 48 h. Ultrathin sections (70 nm) were processed with ultramicrotome (PT-XL PowerTomes, RMC Boeckeler) and placed onto 200 mesh bare copper grids. Samples were stained for 30 min with 4% (w/v) methanol uranyl acetate and 1 min with 0.5% (w/v) aqueous lead citrate. The prepared bacterial samples were examined with a JEM-1400 Plus transmission electron microscope (JEOL USA, Inc.).

### Statistical Analysis

All experiments were conducted in triplicate and the results are expressed as mean ± SD. The obtained data were subjected to a one-way analysis of variance (ANOVA). The statistical program Origin Pro 9.0 was employed for the statistical analysis. The Tukey HSD test was used to identify significant differences (α = 0.05) among the means.

## Results

### The Yield and Composition of Extract

The yield of methanol extract (% dry weight of mushroom) from the *C. versicolor* was 5.68%. The chemical analyses of extract revealed high amount of total phenolics in *C. versicolor*, 25.8 ± 1.4 mg g^-1^ (**Table 1**). Examination of other organic compounds showed that the extract mainly consisted of polysaccharides. The main polysaccharide-containing components in *C. versicolor* methanol extract were β-glucans (197 ± 9 mg g^-1^), while the content of confirmed α-glucans (6 ± 0.5 mg g^-1^) was significantly lower. The content of total proteins in the extract was negligible and amounted to less than 1%.

**Table 1:** Chemical composition^1^ of the methanol extract of *C. versicolor*.

Methanol extract	Total polysaccharide content (mg g^-1^)	Glucan content (mg g^-1^)			Total protein content (mg g^-1^)	Total lipid content (mg g^-1^)	Total phenol content (mg GAE g^-1^)	Total flavonoid content (mg CE g^-1^)
		Total	α	β				
*C. versicolor*	351 ± 19	203 ± 11	6 ± 0.5	197 ± 9	8 ± 0.5	50 ± 2	25.8 ± 1.4	4.3 ± 0.2

*^1^All results are on a dry weight basis of extract and represent mean ± SD (*n* = 3).*

### FTIR Analysis

**FIGURE 1:**
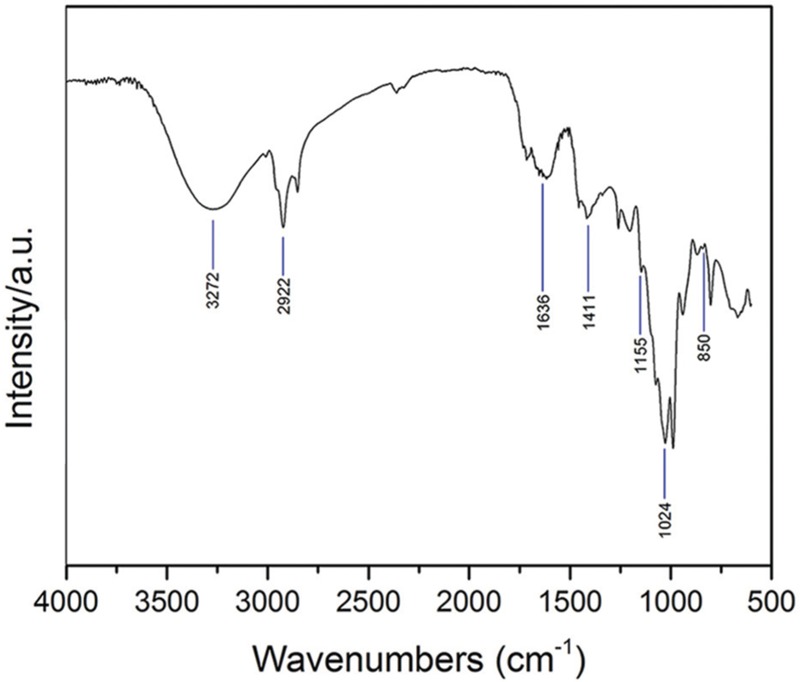
**The FTIR spectra obtained from *C. versicolor* methanol extract**.

Mushroom methanol extract from *C. versicolor* was analyzed using FTIR to determine the chemical composition of the organic compounds. As shown in **Figure 1**, the spectra of mushroom extract indicated a strong broad absorption in the range 3500–3000 cm^-1^ corresponding to the hydroxyl stretching vibration, which could be explained by the molecular interaction of the polysaccharide chains. The presence of asymmetric and symmetric stretching of the N–H bonds in the amino groups was also noted in the same spectral region (Kozarski et al., 2012; Synytsya and Novak, 2014). The two sharp bands at ≈2920 cm^-1^ and ≈2850 cm^-1^ were assigned to asymmetric and symmetric CH_2_ stretching vibrations of lipids, respectively (Mohaček-Grošev et al., 2001; Synytsya et al., 2009). The signal found at ≈1730 cm^-1^ corresponded to the carbonyl stretching vibration of alkyl-esters and indicated the presence of lipids (Mohaček-Grošev et al., 2001; Kozarski et al., 2012). According to these bands, lipids were extracted by methanol together with polysaccharides and some proteins. Three major bands at ≈1640 cm^-1^ (C=O stretching), ≈1560 cm^-1^ (N–H bending) and ≈1350 cm^-1^ (C–N bending) were assigned, respectively, to the amide I, amide II, and amide III vibrations of proteins (Šandula et al., 1999; Synytsya et al., 2009; Synytsya and Novak, 2014). The amide I band could be overlapped by deformation of water near 1640 cm^-1^. Absorption bands between 1410 cm^-1^ and 1310 cm^-1^ were indicative for OH groups of phenolic compounds and the band at ≈1405 cm^-1^ was linked to vibration of aliphatic groups belonging to the phenolic pigments (Kozarski et al., 2012). The absorption bands in the mid-infrared region 1200–800 cm^-1^ (mainly C–C and C–O stretching vibrations in glycosidic bonds and pyranoid rings) indicated the presence of polysaccharides, with different structures and compositions, as the major component. The shoulder near 890 cm^-1^ and bands at ≈1150 cm^-1^, ≈1080 cm^-1^ and ≈1024 cm^-1^ were specific for β-glycosidic bonds and evidenced the presence of β-glucans (Sarangi et al., 2006; Synytsya et al., 2009; Kozarski et al., 2012). The weak bands at ≈850 cm^-1^ and ≈930 cm^-1^ indicated the presence of the α-glycosidic linkages (Šandula et al., 1999; Mohaček-Grošev et al., 2001; Synytsya et al., 2009).

### Broth Microdilution Assay

The results of the antibacterial testing of methanolic mushroom extract are presented in **Table 2**. Extract strongly inhibited the growth of Gram-positive and showed moderate antibacterial activity against Gram-negative bacteria. The most susceptible bacteria was *S. epidermidis* with an MIC value of 0.625 mg mL^-1^ and a MBC value of 1.25 mg mL^-1^. *L. monocytogenes* was the most resistant Gram-positive bacteria with the MIC of 20 mg mL^-1^. The methanol extract from *C. versicolor* acted microbicidal on three out of four assayed Gram-positive bacteria with the concentrations ranging from 1.25 mg mL^-1^ to 5 mg mL^-1^. According to the obtained MIC values, *S. sonnei* was the most sensitive Gram-negative bacteria (MIC = 2.5 mg mL^-1^), followed by *Y. enterocolitica* and *S*. Enteritidis. The highest tested concentration of 40 mg mL^-1^ of the methanol extract of *C. versicolor* acted lethal to *S.* Enteritidis, *Y. enterocolitica*, and *P. hauseri*.

**Table 2:** The antibacterial activity of extract expressed as the MIC (mg mL^-1^) and the MBC (mg mL^-1^) determined by the broth microdilution assay.

Bacterial strain	Source		*C. versicolor* methanol extract
*S. aureus*	ATCC 25923	MIC	2.5 0.0ˆ*
		MBC	5.0 0.0
*S. epidermidis*	ATCC 12228	MIC	0.625 0.000
		MBC	1.25 0.00
*B. cereus*	ATCC 11778	MIC	5.0 0.0
		MBC	5.0 0.0
*L. monocytogenes*	ATCC 19111	MIC	20.0 0.0
		MBC	nd^1^
*E. coli* O157:H7	ATCC 35150	MIC	20.0 0.0
		MBC	*nd*
*S.* Enteritidis	ATCC 13076	MIC	10.0 0.0
		MBC	40.0 0.0
*S. sonnei*	ATCC 29930	MIC	2.5 0.0
		MBC	*nd*
*Y. enterocolitica*	ATCC 27729	MIC	5.0 0.0
		MBC	40.0 0.0
*P. hauseri*	ATCC 13315	MIC	20.0 0.0
		MBC	40.0 0.0

*^∗^Data are expressed as mean ± SD (*n* = 3). ^1^nd, not determined (with the highest tested concentration of samples (40 mg mL^-*1*^), antibacterial activity was not determined).*

### Determination of the Growth Kinetics by Optical Density (OD)

**FIGURE 2:**
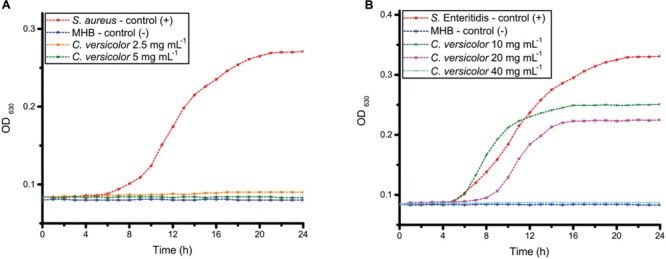
**The growth curves of *S. aureus***(A)** and *S.* Enteritidis **(B)** cells exposed to different concentrations of the methanol extract of *C. versicolor***.

*Staphylococcus aureus* as Gram-positive and *S.* Enteritidis as Gram-negative bacteria were chosen for further antibacterial testing. The growth curves of *S. aureus* and *S.* Enteritidis treated with different concentrations of the methanol extract of *C. versicolor* are shown in **Figure 2**. In presence of 10 and 20 mg mL^-1^ of mushroom methanol extract, the growth curves of *S.* Enteritidis included three phases: lag, exponential and stationary phases. Since the total number of bacteria, including live and dead ones, is assayed by OD_630_, a decline phase could not be detected on the growth curves. The MBC values of extract induced the inhibition of growth which appeared as straight line, parallel to the negative control. Untreated *S.* Enteritidis reached the exponential growth phase rapidly, after about 5 h. When *S.* Enteritidis cells were exposed to 20 mg mL^-1^ of *C. versicolor* methanol extract, the lag phase lasted 7 h before the exponential growth phase began. The concentration 40 mg mL^-1^ of *C. versicolor* methanol extract was previously determined as bactericidal and a bacterial growth phase was not detected on the growth curve when this concentration was used.

Untreated *S. aureus* reached exponential growth phase after about 7 h. The growth curves of *S. aureus* treated with the MIC and the MBC (2.5 and 5 mg mL^-1^, respectively) of *C. versicolor* methanol extract ran parallel with that of the negative control.

### Broth Macrodilution Assay

**FIGURE 3:**
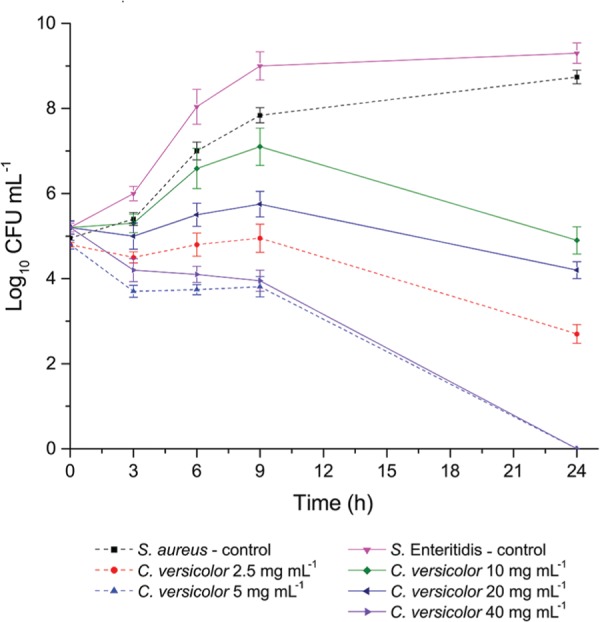
***S. aureus* and *S.* Enteritidis growth, survival and death curves on exposure to methanol extract of *C. versicolor***. Each point represents the log of the mean ± SD CFU mL^-1^.

The growth, survival, and death curves for *S. aureus* and *S.* Enteritidis at various concentrations of *C. versicolor* methanol extract are shown in **Figure 3**. Treatment of *S. aureus* with extract at the MIC (2.5 mg mL^-1^) resulted in an ∼2 log_10_ reduction over 24 h. *S. aureus* showed a 90% decrease of viable count within 3 h of treatment when the MBC (5 mg mL^-1^) was applied and after 24 h all bacteria were killed.

When *S.* Enteritidis was treated with 10 mg mL^-1^ of the extract, after an initial increase of viable count during the first few hours, a subsequent decline of the cells was observed over 24 h. Treatment with 20 mg mL^-1^ of extract resulted in greater than 1 log_10_ reduction. In presence of 40 mg mL^-1^ of extract a constant reduction of viable count was noticed and after 24 h microbicidal effect was established.

### Loss of 260-nm-Absorbing Material

**FIGURE 4:**
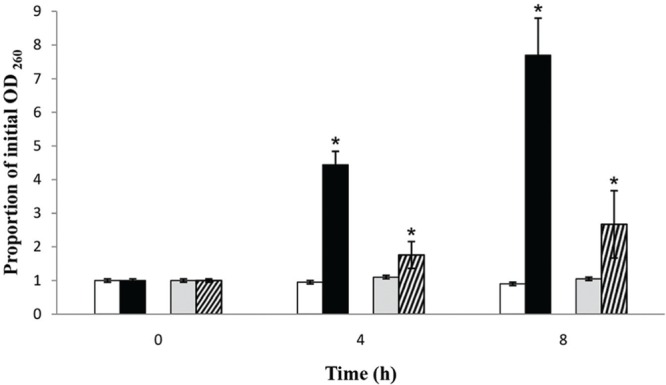
**Presence of 260-nm-absorbing material in the filtrates of *S. aureus* (black bars) and *S*. Enteritidis (bars with diagonal stripes) after treatment with the MIC values of the methanol extract of *C. versicolor* at 4 and 8 h, compared to *S. aureus* (white bars) and *S*. Enteritidis (gray bars) control suspensions.** The mean ± SD for three replicates are illustrated. The asterisk denotes statistical significance (α = 0.05) between the control and the treatments (Tukey’s HSD).

The OD_260_ values of the filtrates from *S. aureus* and *S.* Enteritidis control suspensions remained approximately the same after 4 h as well as after 8 h. Upon treatment with the MIC of the *C. versicolor* methanol extract (**Figure 4**), the OD value of the filtrates from *S. aureus* suspensions drastically increased after 4 h, by about 4.5 times, and especially after 8 h, approximately eight times. The OD value of the filtrates obtained from *S.* Enteritidis treated suspensions increased with time compared with the control, but was markedly lower than the value of the *S. aureus* treated suspensions.

### SEM Observation

**FIGURE 5:**
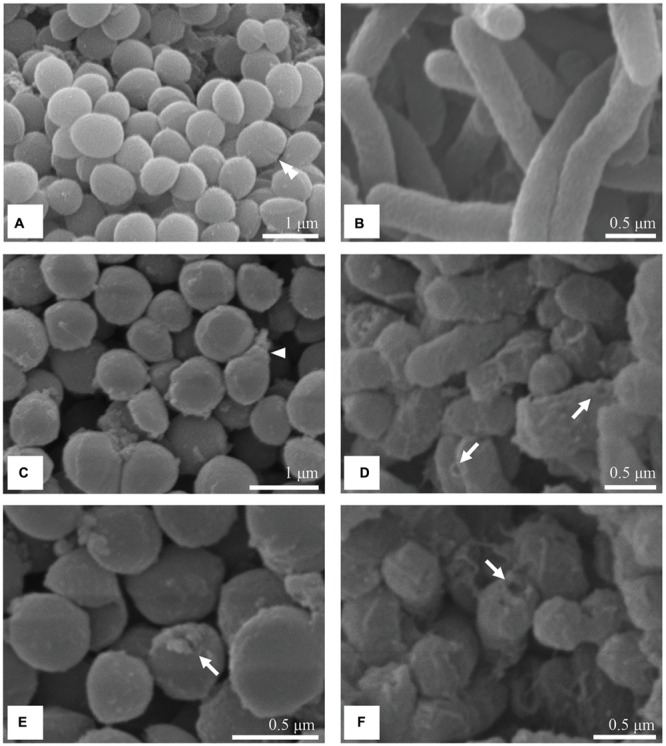
**SEM micrographs of untreated and treated *S. aureus* and *S.* Enteritidis cells.** Untreated cells of *S. aureus* **(A)** and *S.* Enteritidis **(B)** were intact with regular shape and surface. After treatment with the MIC of the methanol extract of *C. versicolor, S. aureus* cells looked misshapen **(C)** and some had holes in their cell wall **(E)**. Treated *S.* Enteritidis cells appeared shorter, aggregated with dimples and blisters on their surface **(D)** and ruptured cell envelope **(F)**.

The untreated cells of *S. aureus* appeared round and without visible abnormalities. The formation of a septum was also observed, which indicated that active proliferation was occurring (**Figure 5A**). After incubation with the MIC of the methanol extract of *C. versicolor*, the bacteria appeared elongated and misshapen (**Figure 5C**) and some had holes in their cell wall (**Figure 5E**).

The control *S.* Enteritidis cells were separated from each other and displayed a smooth and intact surface (**Figure 5B**). The cells treated with the methanolic *C. versicolor* extract appeared shortened with increased compactness, indicating that the cells were not able to reach their full growth (**Figures 5D,F**). In addition, the cells appeared aggregated compared with the control. Moreover, the *S.* Enteritidis cells showed dimples and blisters on their surface, close to the polar and septal regions (**Figures 5D,F**). The rupture of the cell envelope was also noticed (**Figure 5F**).

### TEM Observations

**FIGURE 6:**
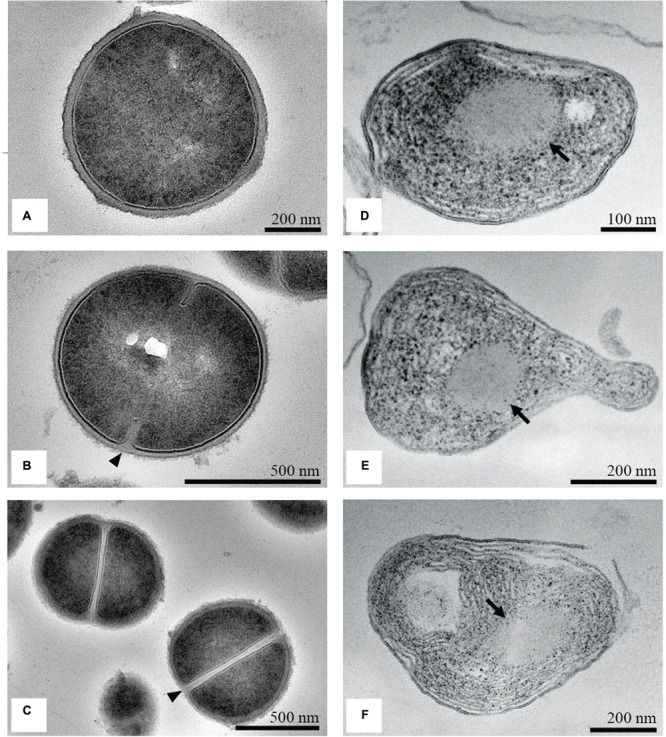
**TEM micrographs of untreated and treated *S. aureus*.** Untreated cells looked round and intact, with a well-defined cell membrane **(A)**. The onset of septation (arrowhead; **B**) and the cross-wall formation (arrowhead; **C**) was noticed. After treatment with the MIC of the methanol extract of *C. versicolor*, cells appeared elongated and malformed, with non-membrane-enclosed bodies (denoted by arrows; **D–F**). Some lysed cells were also noted **(F)**.

The untreated *S. aureus* cells were round, proliferating cells with undamaged cell walls (**Figure 6A**). Cells were captured at different stages in the division process. The growth of wall material into the cytoplasm that indicated the onset of septation, (**Figure 6B**), and cross-wall formation that completely separated the two daughter cells was observed (**Figure 6C**). The intracellular DNA region displayed a relatively homogeneous electron density with a clearly defined cytoplasmic membrane. Near the periphery of the cytoplasm, numerous dark granules corresponding to the ribosomes were detected. After incubation with the MIC of the methanol extract of *C. versicolor*, extensive ultrastructural damages and a wide range of cellular pathologies were observed. TEM micrographs of the treated bacterial cells revealed variation in the cell wall thickness and alteration of the shape, which appeared elongated and malformed compared with the control (**Figures 6D,E**). Additionally, large spherical non-membrane-enclosed bodies with a similar electron density to that of the septal murein layer were observed in it (**Figures 6D–F**). A few lysed cells with severely damaged and depleted cell content and with a lamellar form of the cytoplasm were also noticed (**Figure 6F**).

**FIGURE 7:**
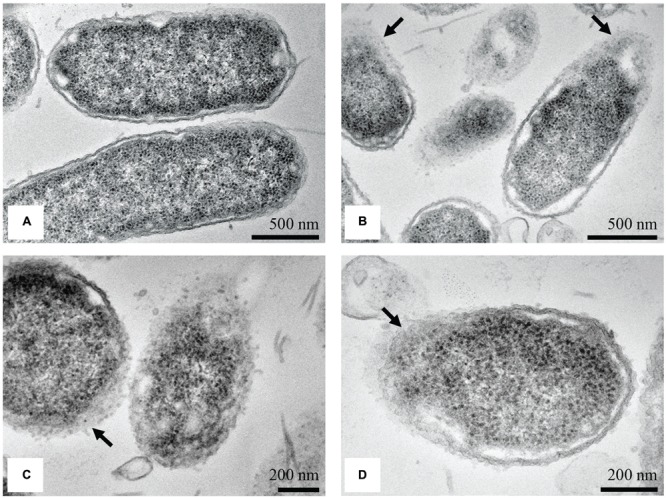
**TEM micrographs of untreated and treated *S.* Enteritidis.** A regular outlined cell envelope and regularly distributed cytoplasm lying closely to the cell wall were observed with untreated cells **(A)**. After treatment with the MIC of the methanol extract of *C. versicolor*, unsymmetrically distributed cytoplasm, larger and irregular periplasmic space and deformed and scattered components of the cell envelop were observed **(B–D)**. The cells without the membranes **(C)** as well as null cells **(D)** were also noted.

Transmission electron microscopy micrographs of untreated *S.* Enteritidis cells showed a regular rod-shaped structure with an undamaged and slightly waved outer membrane. The intracellular content was well-maintained with the cytoplasmic membrane lying close to the cell wall. The periplasmic space was thin with a homogeneous appearance while the intracellular region displayed a heterogeneous electron density (**Figure 7A**). After treatment with the MIC of the methanol extract of *C. versicolor*, multiple changes in the cell morphology were observed. The periplasmic space appeared hyperhydrated with significantly increased an electron-lucent region, suggesting detachment of the cell wall from the plasma membrane. The components of the bacterial cell envelope were deformed and scattered from their original form and the intracellular structures were disorganized (**Figure 7B**). At certain locations of the cells, mostly the septal and polar regions, a draining out of the intracellular contents due to rupture of the cell envelope was noted. Null cells were found (**Figure 7D**) and many cells without membranes (**Figure 7C**).

## Discussion

Numerous mushroom extracts were reported as having a more pronounced antimicrobial activity against Gram-positive compared with Gram-negative bacteria (Yamaç and Bilgili, 2006; Venturini et al., 2008; Karaman et al., 2010; Alves et al., 2012), which is in agreement with the results obtained in this study. Methanol extract of *C. versicolor* inhibited the growth of *Bacillus* sp. (Karaman et al., 2010), while *S. aureus* was sensitive to the methanol extracts, as well as to the ethyl acetate, dichloromethane, and acetone extracts (Yamaç and Bilgili, 2006; Karaman et al., 2010). Chu et al. (2002) reported that a sesquiterpene, coriolan, isolated from species of *Coriolus*, inhibits the growth of Gram-positive bacteria. Additionally, Alves et al. (2013) tested the antimicrobial activity of phenolic compounds identified in mushrooms. *S. aureus, S. epidermidis*, and *L. monocytogenes* were inhibited by protocatechuic and caffeic acid, detected in *C. versicolor* (MIC = 1 mg mL^-1^). The results obtained in the present study showed that Gram-positive bacteria were sensitive to *C. versicolor* methanol extracts. The established MIC values for *Staphylococcus* sp. and *B. cereus* varied from 0.625 to 5 mg mL^-1^ for *C. versicolor* extract.

According to literature data, the Gram-negative bacteria *E. coli, S. flexneri*, and *P. mirabilis* were resistant, while *S.* Enteritidis was highly sensitive to the methanol extract of *C. versicolor* (Karaman et al., 2010). *P. aeruginosa* was inhibited only by the acetone extract of the same mushroom, but the established inhibition zones (<10 mm) indicated its weak effect (Yamaç and Bilgili, 2006). In the present study, higher concentrations of methanol extract were required to inhibit the growth of Gram-negative bacteria (MIC values in the 2.5 to 20 mg mL^-1^) in comparison to Gram-positive.

An explanation for the different sensitivity among the strains could be the fact that the peptidoglycan of Gram-negative bacteria is surrounded by an outer membrane, which restricts diffusion through its lipopolysaccharide (LPS) covering. Genetic and chemical experiments proved that the LPS layer plays an essential role in providing selective permeability (Li et al., 2010) and serves as an efficient barrier against rapid penetration of various compounds (harmful agents). Moreover, the periplasmic space of Gram-negative bacteria contains enzymes (such as hydrolytic and detoxifying) which are able to inactivate foreign molecules introduced from the environment. On the other hand, Gram-positive bacteria lack the outer membrane but are characterized by thick, hydrophilic, porous structure that makes them more permeable. Therefore, Gram-positive bacteria are expected to be more sensitive to exposure to mushroom extracts (Ren et al., 2014; Duvnjak et al., 2016).

Even though some strains showed lower MIC values, *S. aureus* and *S.* Enteritidis were chosen for further antibacterial testing. Both, non-typhoidal *Salmonella* spp. and *S. aureus* are extremely adaptable pathogens, notorious for their ability to develop resistance to antibiotics (CDC, 2013). The total medical cost of antibiotic resistance to the world economy ranges in billion. The changing pattern of resistance in *S. aureus* and decreasing efficacy of existing drugs have highlighted the necessity for new antistaphylococcal agents (Lowy, 2003; Chambers and DeLeo, 2009). According to the 2011 estimates, the most common bacterial foodborne illnesses, hospitalizations and deaths with tens of millions of human cases occurring worldwide every year are caused by Gram-negative non-typhoid *Salmonella* spp. and *S. aureus* as Gram-positive bacteria (CDC, 2011). The diseases caused by *Salmonella* spp. are a public health concern globally, in high- and low-income countries alike (WHO, 2015). The results obtained by the broth microdilution and macrodilution assays and by measuring the growth kinetics at 630 nm indicated that the *C. versicolor* methanol extract inhibited the growth of the tested bacteria. The curves of *S.* Enteritidis obtained in presence of 10 and 20 mg mL^-1^ were slightly different to those established using microplate reader and the reason is most likely the inability of the device to distinguish dead from live cells. According to these data, mushroom methanol extract demonstrated promising antibacterial activity.

Methanolic extracts of mushrooms are widely recognized as being rich in polyphenols. Cheung and Cheung (2005) found that the majority of the low molecular weight compounds from mushrooms (e.g., phenolic fractions) were extracted by methanol. In the same study, the high polarity of the chemical components in methanol extracts was noted. Using RP-HPLC analysis, Harhaji et al. (2008) evaluated the composition of the methanol extract of *C. versicolor* and found numerous components characteristic for phenols and terpenoids. In this study, the content of total phenolics (25.8 ± 1.4 mg g^-1^) was in accordance with previously published data. Mau et al. (2002) established a content of total phenolics of 23.3 mg g^-1^ in *C. versicolor* methanol extract. Generally, there is a lack of information about chemical composition of *C. versicolor* extracts. The results obtained in this study indicated higher content of total phenols and flavonoids than in the *C. versicolor* water and ethanol extracts, 18–22.4 mg g^-1^ and 3.36 mg g^-1^, respectively (Vidović, 2011; Kozarski et al., 2012). The total polysaccharides, total glucans and total proteins were found in higher amount in water and ethanol extracts by the same authors (724–839 mg g^-1^, 363 mg g^-1^, and 39–159 mg g^-1^, respectively). The low content of proteins, obtained in this study, was probably due to use of methanol as the extraction solvent since it may cause their denaturation and precipitation.

Comparing the chemical composition of the extracts, only phenolic compounds were found in higher amounts in the methanol extract of *C. versicolor*, which indicated that they were, most likely, responsible for the high antimicrobial activity. According to previously reported data, Gram-positive bacteria are more susceptible to polyphenols than Gram-negative bacteria (Cardona et al., 2013). In addition, hydroxycinnamic acids (*p*-coumaric, caffeic and ferulic) induce greater potassium and phosphate leakage than hydroxybenzoic acids (protocatechuic, gallic, and vanillic; Campos et al., 2009). Karaman et al. (2010) examined the content of phenolic acids in the methanol extract of *C. versicolor* and detected higher amount of caffeic acid compared with gallic and protocatechuic. It is assumed that phenolic acids, due to their partially lipophilic nature, pass through the cell membrane by passive diffusion and cause an increase in membrane permeability. They possibly reduce the intracellular pH and induce protein denaturation. When Gram-positive *S. aureus* cells were treated with the MIC of *C. versicolor* methanol extract, SEM revealed elongated and malformed cells while pronounced leakage of intracellular materials was observed at 260 nm. These observations suggested that major and irreversible damage and loss of permeability of the cytoplasmic membrane had occurred. It was reported that essential oils and their components (terpenes and their related alcohols) exhibit antimicrobial activity against a variety of cell parts, particularly the membrane and cytoplasm, and in some cases, completely change the morphology of the cells (Carson et al., 2002; Nazzaro et al., 2013). The altered surface morphology of *S. aureus* was noticed when cells were treated with *Origanum vulgare* essential oil, which indicated that the cytoplasmic membrane was compromised (de Souza et al., 2010). In addition, many established antimicrobial agents that act on the cytoplasmic membrane induce the release of extracellular UV-absorbing material, including polymyxin, tetracyclines, α-pinene, lemongrass oil, tea tree oil, and eugenol (Carson et al., 2002; Devi et al., 2010). TEM micrographs of treated cells corroborated the inability of the methanol extract of *C. versicolor* to lyse *S. aureus* and suggested membrane damage by the appearance of spherical non-membrane-enclosed bodies and a loss of cytoplasmic material. Hartmann et al. (2010) reported the presence of spherical non-membrane-enclosed bodies with an electron density similar to that of the septal murein layer in *S. aureus* cells treated with AMPs. They assumed that such bodies were formed in the cytoplasm when translocation of lipid-linked precursors from the cytoplasmic side to the other side of the membrane was disabled and represented the accumulated peptidoglycan and teichoic acid precursors. It is known that newly formed peptidoglycan is inserted only at the division septum in coccoid cells like those of *S. aureus* (Scheffers and Pinho, 2005). In the present study, the formation of septa in the treated sample in comparison with the control was not observed. Therefore, it could be assumed that exposure to methanol extract of *C. versicolor* disables cell division (i.e., the formation of septa) and instead leads to the accumulation of peptidoglycan and teichoic acid precursors in the cytoplasm. Since the biosynthesis and cross-linking of peptidoglycan are functions regulated by the cytoplasmic membrane, this observation also indicates membrane damage. Although, a few cells with holes in their cell wall (**Figure 5E**) and lysed cells were noticed, their inner content already looked severely damaged (**Figure 6F**). The lysed cells were presumably found because of the long incubation period of the cells with the *C. versicolor* extract (8 h) before they were fixed in glutaraldehyde. The lysis probably occurred either due to activation of membrane–bound cell wall autolytic enzymes or as the consequence of weakening of the cell wall.

In the case of the Gram-negative *S.* Enteritidis cells treated with the MIC of *C. versicolor* methanol extract, SEM showed shortened and aggregated cells with dimples and blisters on their surface and ruptured cell walls. The dimples and blisters, distinct signs of damage to the cell envelope, were observed mostly at the polar and septal region. This location might be explained by the interaction of the methanol extract of *C. versicolor* with the negatively charged cardiolipin-rich domain, which is concentrated at the poles of bacterial cells (Romantsov et al., 2009). Furthermore, aggregation of the cells probably occurred as a result of the draining out of cytoplasmic material through the damaged cell wall. The aggregation of *S.* Enteritidis, which appeared as a sludge, was noticed when the cells were treated with methanolic and ethanolic *Porella arboris-vitae* extracts that acted by interfering with the outer cell walls of the bacterial cells (Tyagi et al., 2013). Furthermore, rough surface morphology and shrinkage of treated cells was observed when *Salmonella* Typhi and *E. coli* were treated with eugenol and AMPs, respectively. Both antimicrobial agents acted by damaging the cell membranes (cell envelope) of the bacteria (Devi et al., 2010; Hartmann et al., 2010). In this study, the leakage of 260-nm-absorbing material from the cell interior, which is indicative of gross membrane damage, was less pronounced in *S.* Enteritidis than in *S. aureus* treated cells. These results suggested that the primary target of the methanol extract of *C. versicolor* was not disturbance of the cytoplasmic membrane, but damage of the cell envelope. TEM micrographs revealed scattered and deformed components of the bacterial cell envelope, mostly at the septal and polar regions. Moreover, null cells and cells without membranes were noticed, indicating that the complete cytoplasmic content had leaked out of the cells. Since the outer membrane of Gram-negative bacteria consists of LPSs and membrane proteins, it could be assumed that they were the potential target sites on which the methanol extract of *C. versicolor* acted.

According to the obtained results, it can be concluded that the methanol extract of *C. versicolor* primary acted on the cytoplasmic membrane of *S. aureus* while direct damage of the bacterial cell envelope of *S.* Enteritidis was revealed. The different behavior was probably the consequence of the different structures of the cell wall between Gram-positive and Gram-negative bacteria. Since the *C. versicolor* extract, and mushroom extracts in general, are a complex mixture of various compounds, it is highly probable that the components responsible for their antimicrobial activity are not the same for Gram-positive and Gram-negative bacteria. The use of crude extracts is presumably the most desired, since any purification leads to reduction of their biological activity. In this study, the FTIR spectra of the tested methanol extract indicated that the antimicrobial activity of *C. versicolor* was most probably related to the diverse chemical composition of its extract. Additionally, given the heterogeneous composition of the extract, the possibility of the existence of more than one target site should not be ruled out.

The examined bacterial strains have been mainly recognized as agents of food poisoning, and the use of *C. versicolor* extract as potential preservative might help to reduce the contamination and to extend the shelf life of food. In addition, based on the moisture content of mushroom, the yield of methanol extract and the obtained effective concentrations, the total amount of fresh fruiting bodies needed to inhibit bacterial growth was calculated. The determined weight of the wet starting material ranged from 0.05 to 2.35 g. The present results suggest that a potential application of *C. versicolor* methanol extract, as natural food preservative, could also have positive economic outcome. Practical implementation in the food industry would certainly be profitable considering the cheap substrates required for mushroom growth. However, the actual economic aspects could be calculated only after the testing of the extracts in a real food system. Further work is required to fully understand the mechanisms involved in antimicrobial activity of mushroom extracts with the aim of finding new natural antimicrobial agents. After performing cytotoxicity assays and elucidation of the mode of action, research should also be focused on testing the antimicrobial activity of extracts in food industry, as well as their interaction with the food components.

## Author Contributions

DM and MP conceived and designed the research and interpreted the data; DM, MP, BR, DD, and VP contributed to acquisition and analysis of data; DM, MP, AS, and VP drafted the manuscript; MN contributed to the conception of the work and did final approval of the version to be published.

## Conflict of Interest Statement

The authors declare that the research was conducted in the absence of any commercial or financial relationships that could be construed as a potential conflict of interest.
